# Role of tandospirone, a 5-HT1A receptor partial agonist, in the treatment of central nervous system disorders and the underlying mechanisms

**DOI:** 10.18632/oncotarget.22170

**Published:** 2017-10-27

**Authors:** Xuefei Huang, Jing Yang, Sijin Yang, Shousong Cao, Dalian Qin, Ya Zhou, Xiaoli Li, Yun Ye, Jianming Wu

**Affiliations:** ^1^ Department of Clinical Pharmacy, School of Pharmacy, Southwest Medical University, Luzhou, Sichuan, 646000, China; ^2^ Laboratory of Chinese Materia Medica, Department of Pharmacology, School of Pharmacy, Southwest Medical University, Luzhou, Sichuan, 646000, China; ^3^ Department of Cardiology and Neurology, Affiliated Traditional Chinese Medicine Hospital, Southwest Medical University, Luzhou, Sichuan, 646000, China; ^4^ Laboratory of Cancer Pharmacology, Department of Pharmacology, School of Pharmacy, Southwest Medical University, Luzhou, Sichuan, 646000, China; ^5^ Department of R&D, Sichuan CREDIT Pharmaceutical Ltd., Luzhou, Sichuan, 646000, China; ^6^ Department of Pharmacy, Affiliated Hospital of Southwest Medical University, Luzhou, Sichuan, 646000, China

**Keywords:** tandospirone, 5-HT1A receptor, Parkinson's disease, schizophrenia, mechanisms

## Abstract

5-hydroxytryptamine (5-HT, serotonin) is an important neurotransmitter in the modulation of the cognitive, behavioral and psychological functions in animals and humans. Among the fourteen subtypes of 5-HT receptor, 5-HT1A receptor has been extensively studied. Tandospirone, an azapirone derivative with strong and selective agonist effect on 5-HT1A receptor, has been used for the treatment of anxiety disorders especially generalized anxiety disorder for decades. Recently, tandospirone showed the efficacy in relieving the syndromes of social anxiety disorder and post-traumatic stress disorder as well as in potentiating the effect of antidepressants in the treatment of depression in both preclinical and clinical studies. More impressively, the beneficial effect of tandospirone has been revealed on improvement of motor dysfunction of Parkinson's disease and cognitive deficits of schizophrenia either in monotherapy or in combination with other drugs. This review discusses the superiority of tandospirone in the treatment of the disorders and associated mechanisms in central nervous system from the literature.

## INTRODUCTION

5-hydroxytryptamine (5-HT, serotonin), a biogenic amine, acts as a neurotransmitter and is discovered in wide variety of sites in the central and peripheral nervous system [1]. In brain, 5-HT neurons are mainly located in the raphe nuclei, whose relatively small number of neurons by projections innervate almost all brain areas as diverse as the limbic areas, cerebral cortex, basal ganglia, diencephalon and spinal cord. Thus, 5-HT binds to different receptors to execute significant effects on generation and modulation of cognitive, behavioral and psychological functions in the central nervous system (CNS) in animals and humans [2, 3].

The distinct receptors of 5-HT comprise seven main families (5-HT1 to 5-HT7) with at least fourteen subtypes on the basis of their pharmacological responses to specific ligands, sequence similarities at gene and amino acid levels, gene organization and second messenger coupling pathways [4, 5]. Except for 5-HT3 receptor, which is assigned into the ionotropic receptor family, the rest of the hitherto identified receptors (5-HT1-2, 5-HT4-7) belong to the seven-transmembrane domain G-protein coupled receptor (GPCR) family [6]. Among these receptors, 5-HT1A receptor is thought to be the most important and has been extensively studied.

## 2 5-HT1A RECEPTOR AND ITS AGONISTS

### 5-HT1A receptor

The 5-HT1A receptor is the first subtype to be cloned and sequenced among all the serotonin receptors [7]. Analyzing the sequence of this genomic clone showed that 50% of amino acids were homologous with the β2-adrenergic receptor in the transmembrane domain [8]. Furthermore, the 5-HT1A receptor gene is located on human chromosome 5q11.1-q13 and the encoded protein consists of 421 amino acids in human and mice while 422 amino acids in rats [9, 10]. More importantly, it was accessible to visualize the sites of 5-HT1A receptor in various regions of brain at the sub-cellular level by the polyclonal antibodies [11]. The 5-HT1A receptor has been detected in limbic forebrain regions (e.g. hippocampus, raphe nuclei, amygdala) with high density, while in extrapyrimidal areas (e.g. basal ganglia, substantia nigra) with low density [12]. They are present on the soma and dendrites of 5-HT neurons isolated from raphe nuclei as presynaptical autoreceptors to inhibit the firing rate of 5-HT, and on postsynaptic neurons such as hippocampus and amygdala innervated by 5-HT neurons as heteroreceptors, where they also attenuate firing activity [13].

Since the crystal structures of 5-HT1B and 5-HT2B receptors have been well studied [14], a homology model of 5-HT1A receptor using the crystal structure of the 5-HT1B receptor (PDB ID: 4IAQ) was established to explore the structure basis of the stereoselectivity of a prototypical GPCR [15]. Using molecular interaction fingerprints, it was discovered that the agonist of 5-HT1A receptor could mobilize nearby amino acid residues to form a continuous water channel via molecular switches, while the antagonist of 5-HT1A receptor maintained stabilization in the binding pocket [16]. Although the accurately targeted site of 5-HT1A receptor by tandospirone is still unknown, it is rational to speculate that as a partial agonist of 5-HT1A receptor, tandospirone may act through forming a continuous water channel by mobilizing nearby amino acid residues.

### 5-HT1A receptor agonists

5-HT1A receptor agonists are one sort of the ligands which is able to activate the 5-HT1A receptors. According to different intrinsic activities, 5-HT1A agonists are clarified in two categories, namely full agonists such as 8-OH-DPAT, F-11440 and flesinoxan, as well as partial agonists such as ipsapirone, gepirone, buspirone and tandospirone [17–20]. Tandospirone is highly potent among partial agonists of 5-HT1A receptor and has a *K*_i_ value of 27 ± 5 nM. Moreover, tandospirone is approximately two to three orders of magnitude less potent at 5-HT2, 5-HT1C, α1-adrenergic, α2-adrenergic and dopamine D1 and D2 receptors (*K*_i_ values ranging from 1300 to 41000 nM) than at 5-HT1A [21]. Taken together, unlike other azapirones such as buspirone and ipsapirone with moderate-to-high affinity for the dopamine D2 receptor and α1- adrenergic receptors, respectively, tandospirone has a potent and selective agonist effect on 5-HT1A receptor [22, 23]. Specifically, tandospirone is characterized as a full agonist at 5-HT1A autoreceptors in the raphe nuclei as well as a partial agonist at postsynaptic 5-HT1A receptors in the forebrain areas receiving 5-HT input [24, 25].

Tandospirone Citrate (Sediel) was firstly synthesized by Dainippon Sumitomo Pharmaceuticals in 1980 and marketed in 1996 in Japan. It was available in China in 2004. In both countries, tandospirone was permitted for the treatment of anxiety disorder especially generalized anxiety disorder and anxiety associated with primary hypertension or peptic ulcer. Besides, tandospirone showed the efficacy in treating other CNS disorders such as depression, Parkinson's disease and schizophrenia in recent clinical and preclinical studies.

## TANDOSPIRONE IN THE TREATMENT OF CNS DISORDERS

### Tandospirone in the treatment of anxiety disorders

Anxiety disorders are the most common psychiatric disorders with lifetime prevalence up to 14%, and have enormous burden on both society and individuals [26–30]. To date, there are seven recognized anxiety syndromes: panic disorder, agoraphobia, social anxiety disorder (SAD), generalized anxiety disorder (GAD), specific phobias, obsessive compulsive disorder (OCD) and post-traumatic stress disorder (PTSD). Pharmacological treatments for anxiety disorders are benzodiazepines, tricyclic antidepressant drugs, selective serotonin reuptake inhibitors (SSRIs), serotonin and noradrenaline reuptake inhibitors (SNRIs) and partial 5-HT1A receptor agonists. Because of low selectivity to diverse receptors, benzodiazepines (BZP) exert a series of adverse effects such as sedation, muscle relaxation, dependence and cognitive impairment [31, 32]. SSRIs, a first-line treatment for many anxiety disorders, have received reduced acceptability in clinical practice due to a delayed onset of action, possible side effect of sexual dysfunction and even transiently increased anxiety [33].

**Figure 1:**
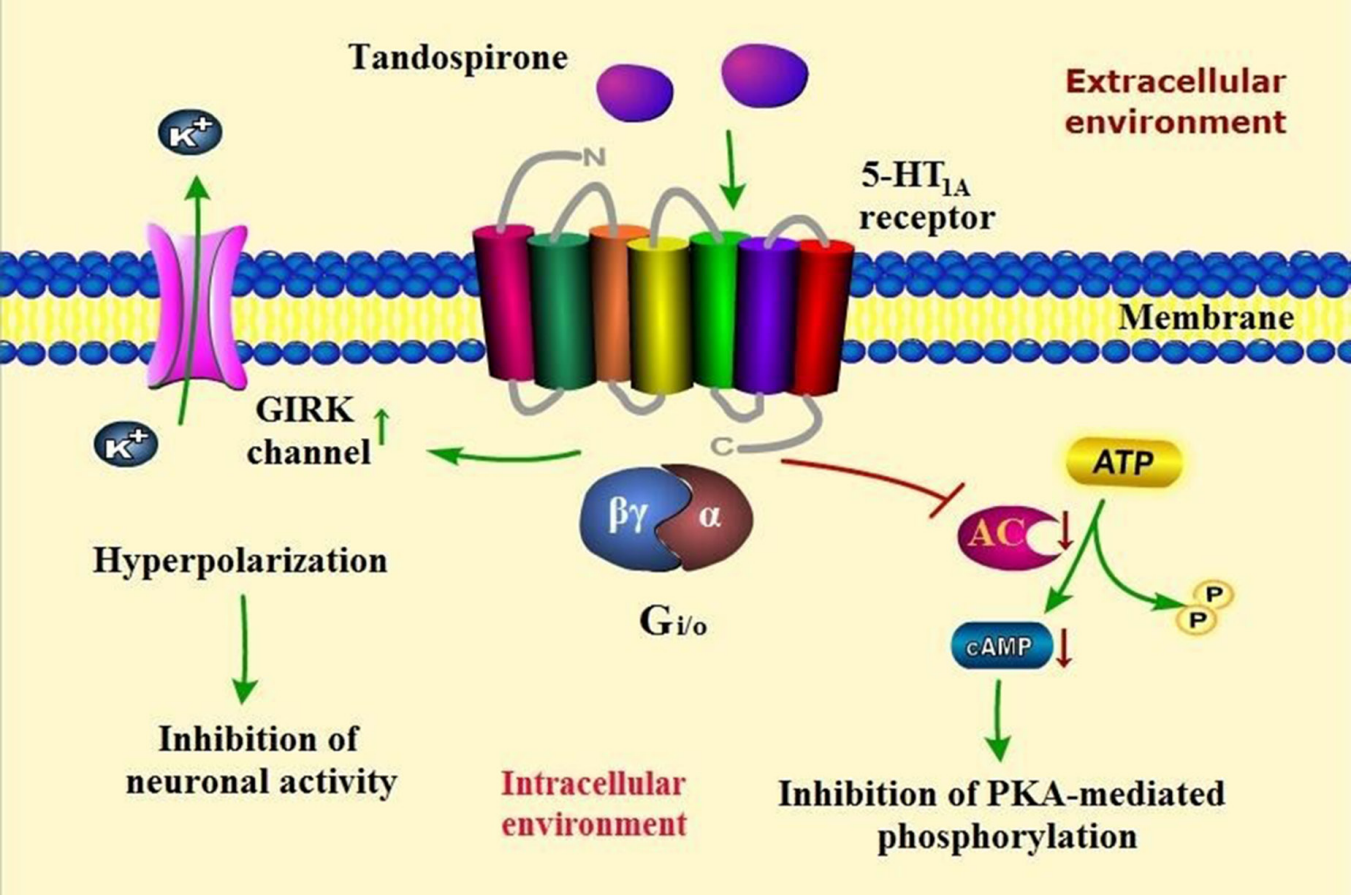
A proposed scheme of tandospirone and its signal transduction pathway in the treatment of anxiety disorders Tandospirone activats postsynaptic 5-HT1A receptor coupled with G-protein (G_i/o_), resulting in inhibition of protein kinase A (PKA)-mediated protein phosphorylation and neuronal activity.

Tandospirone showed anxiolytic action in variety of animal models [25, 34] and has been widely used in treatment of anxiety disorders especially in GAD [13, 35, 36]. Because of high selectivity to 5-HT1A receptor, tandospirone exerts definitely anxiolytic effect without benzodiazepines-like side effects such as sedation and muscle relaxation. Although the role of presynaptic 5-HT1A receptor is still uncertain, tandopirone shows its anxiolytic profile by activating postsynaptic 5-HT1A receptor coupled with G-protein (G_i/o_). On the one hand, tandospirone inhibits the activity of adenylate cyclase by coupling with G_iα_, resulting in reduction of cAMP formation and consequently inhibition of protein kinase A (PKA)-mediatied protein phosphorylation. On the other hand, it activates G-protein-gated inwardly rectifying potassium (GIRK) channels by releasing G_βγ_ subunits, leading to efflux of intracellular K^+^, hyperpolarization of targeted neurons and ultimately inhibition of neuronal activity [37–39]. As described above, postsynaptic 5-HT1A receptors are mainly located in the limbic areas such as hippocampus, amygdala as well as septum. Furthermore, tandospirone is capable of inhibiting the firing of hippocampal and lateral septal neurons by activating GIRK channels [24, 40, 41]. Thus, it may be anticipated that tandospirone alleviates anxiety through inhibiting the activity of hippocampus and amygdala which are associated with the induction of anxiety as well as the lateral septum referring to anxiety transmission. (Figure 1) However, short half-time and one- or two-week delayed onset are the major disadvantages of tandospirone in the treatment of anxiety [40].

Studies with double-blind, randomized clinical trials have proven that oral administration of tandospirone was equivalently effective as oral diazepam or clonidine in reducing preoperative anxiety in patients with elective otolaryngologic surgery [42, 43]. In the rat contextual conditioned fear stress model, tandospirone or SSRIs (e.g. paroxetine, fluvoxamine, citalopram) significantly inhibited the conditioned freezing in a dose-dependent manner, respectively, and the combination therapy was addictive for fluvoxamine facilitating anxiolytic effect of tandospirone via CYP3A4 inhibition [44–46]. Moreover, tandospirone exhibited therapeutic potential for facilitating fear extinction to alleviate anxiety, and the effect was mediated by enhancing cortical dopamine neurotransmission indirectly *via* the ventral tegmental area (VTA)-hippocampus dopaminergic loop and improving synaptic efficacy in the extinction processes in the animal model of PTSD [47, 48]. More recently, tandospirone has been proven to be safe and effective and it appeared non-inferior to sertraline for treating SAD in youths in an eight-week randomized open-label trial [49]. Taken together, tandospirone may be an alternative agent in relieving anxiety in the treatment of PTSD and SAD.

### Tandospirone in the treatment of depression

Depressive disorder (major and minor) is a chronic, highly recurrent, and debilitating mental disease with highly suicide rate and has a lifetime prevalence of up to 20% [50]. Depression was the leading cause of disability globally by a recent WHO announcement [51]. Over the past 50 years, pharmacological approaches for the treatment of depression have updated from tricyclic antidepressants and monoamine oxidase inhibitors, to SSRIs and SNRIs. Nonetheless, these advances are far from optimistic because of suboptimal treatment response and low remission rates. For instance, the pooled response rates were 37% and 54% for placebo and drug, respectively, in a meta-analysis of 182 antidepressant randomized controlled trials (RCTs, n = 36,385) [52].

The dysfunction of 5-HT system, such as 5-HT deprivation, has been widely accepted to play a crucial role in the pathogenesis of depression [53]. Tandospirone had shown its antidepressant effect in several forced swimming tests in an animal model of depression [54–57]. Acute administration of tandospirone decreased the releasing of 5-HT in the nerve terminal region, the dendrites and cell body region, while chronic treatment induced desensitization of somatodendritic 5-HT1A autoreceptors, relieving 5-HT neurons from autoreceptor-mediated self-inhibition, eventually tonically activating of 5-HT neurons and counteracting the serotonergic deficit. On the other hand, the sensitivity of postsynaptic 5-HT1A receptors was non-altered even after repeated treatment of tandospirone [13, 40]. It is putative that the duration of desensitizing of presynaptic 5-HT1A autoreceptors in the raphe nuclei is sufficient for tandospirone's antidepressant activity, and it also explains the late onset of action of tandospirone treatment. Several clinical studies have proven that co-treatment of tandospirone and SSRIs synergistically facilitated the desensitization of 5-HT1A autoreceptors, thus exhibiting a more rapid onset of action and/or augmenting the antidepressant actions [44, 58, 59]. Furthermore, an increasing body of research evidence has shown that the antidepressant activity of tandospirone may be associated with other pharmacodynamics effect. Clinical studies showed that tandospirone potentiated the efficacy of fluoxetine, a SSRI, in the treatment of major depressive disorders. In animal studies, tandospirone augmented fluoxetine-induced increase in extracellular dopamine level in dialysates of medial frontal cortex in rat with 200% or 380% of basal levels for fluoxetine alone or for fluoxetine in combination with tandospirone, respectively [60]. Furthermore, increased hippocampal neurogenesis is implicated in the action mechanism of antidepressants [61]. In two recent studies, chronic treatment of tandospirone reversed the decrease in the density of doublecortin (DCX)-positive cells, a marker protein of newborn neurons, in the dentate gyrus of hippocampus in intermittent social defeat rat or directly increased the number of the DCX-positive cells in normal rats, indicating that chronic tandospirone treatment exerted antidepressant action also via increasing hippocampal neurogenesis [57, 62]. In terms of energy metabolism, the footshock stress-induced increment of extracellular lactate concentrations in the prefrontal cortex of rats was suppressed by chronic treatment of tandospirone, but it is still in debate whether this effect of tandospirone is related to ameliorating anxiety and depression or not [63].

A randomized, controlled clinical trial for evaluation of the efficacy of clomipramine alone and in combination with tandospirone or diazepam for 6 weeks in 36 untreated outpatients with major depressive disorder was conducted, and no statistically significant differences in improvement of major depressive disorders were observed in the terms of the Hamilton Depression Rating Scale and the Hamilton Anxiety Rating Scale scores among the three groups. However, tandospirone may induce early antidepressant effects in augmentation of clomipramine [64]. Given the small sample capacity, a larger randomized controlled trial is needed to confirm the conclusion. Besides, the superiority of tandospirone in the treatment of depression also lies in following circumstances. Primarily, antidepressants such as tricyclic antidepressants with anticholinergic activity may induce severe intestinal dysfunction and delirium, while monotherapy of tandospirone significantly ameliorated the depressive mood, agitation and anxiety of senile patients with dementia with little anticholinergic activity [65]. Secondly, eighty to ninety percent of patients with major depression are suffering from sleep disturbance, which is widely accepted as a risk factor and/or prodromal symptom of depression [66–69], while tandospirone could markedly improve sleep disturbance induced by Adrenocorticotropic hormone (ACTH) in a rat model [70]. Finally, since SSRIs are still the first choice for the treatment of patients with depression, the side effect of SSRIs could not be ignored. A case report in Japan showed systematic administration of tandospirone could effectively treat paroxetine-induced bruxism by increasing dopamine release in the prefrontal cortex [71].

### Tandospirone in the treatment of Alzheimer's disease

Alzheimer's disease (AD) is one of the most common neurodegenerative diseases and characterized by impairments in cognition and behavior, eventually resulting in decline in activities of daily living. Nearly 80% of patients with AD have suffered behavioral and psychological symptoms of dementia (BPSD) in the course of the illness. The International Psychiatric Association defines Behavioral and Psychological Symptoms of Dementia (BPSD) as non-cognitive symptoms such as behaviors (e.g. agitation, aggression, wandering, and screaming) and psychiatric disturbances (e.g. hallucination, delusion, depression, anxiety, and insomnia). Classical drug therapy for AD consists of acetylcholinesterase (AChE) inhibitors (e.g. donepezil and galantamine etc.) and N-methyl-D-aspartate (NMDA) receptor antagonist (e.g. memantine), these drugs have shown moderate efficacy in relieving cognitive and functional symptoms. However, the biggest challenge for AD treatment is to discover and develop novel drugs that could substantially slow disease progression or even cure the disease. For the treatment of BPSD, common psychotropic agents and atypical neuroleptics only have moderate efficacy with increased mortality [72], therefore, it is imperative to develop safer and more effective agents.

Together with dysfunction of cholinergic and glutamatergic system, abnormal serotonergic system is also thought to contribute to learning and memory impairment in AD patients. It was found decreased radioligand [^11^C]WAY100635, a potent antagonist of 5-HT1A receptor, binding to 5-HT1A receptor in hippocampus and cortex in patients with AD compared with healthy volunteer. On the other hand, there were negative correlations between 5-HT1A density and aggressive behavior of AD patients from a post-mortem study [73]. Taken together, 5-HT1A receptor antagonists were speculated to be effective for the treatment of memory impairment in AD patients [74]. However, none of such 5-HT1A receptor antagonists were available for clinical use up to date. A preliminary open-label study showed that tandospirone was effective in the treatments of BPSD in thirteen outpatients with DSM-IV of AD or vascular dementia [75]. Significant improvement of psychological symptoms such as depression, anxiety and irritability/lability and behavioral symptoms such as agitation and aggression without serious adverse effects were observed, suggesting that tandospirone is a promising medicine for the treatment of BPSD with remarkable tolerability and improvement of cognitive and memory deficits, and its effectiveness may not only be simply correlated with the density or binding potential to 5-HT1A receptors [75].

### Tandospirone in the treatment of Parkinson's disease

Parkinson's disease (PD) is a prevalent, late-onset neurodegenerative disease, which is due to lesions of nigro-striatal dopamine neurons or depletion of dopamine. PD has a wide spectrum of clinical features including extrapyramidal motor symptoms (e.g. akinesia/bradykinesia, tremor, rigidity and postural defect) as well as various non-motor symptoms (e.g. cognitive impairment, mood disorders, autonomic dysfunction and sleep disorders) [76]. After nearly 40 years, the dopamine precursor L-3,4-dihydroxyphenylalanine (levodopa or L-DOPA) is still the golden-standard treatment for PD. Other antiparkinsonian agents are dopamine D2/D3 agonists (e.g. bromocriptine, cabergoline, pramipexole), dopamine releasers (e.g. amantadine), along with muscarinic acetylcholine (mACh) receptor antagonists (e.g. trihexyphenidyl and biperidene). Inhibitors of monoamine oxidase-B (MAO-B) (e.g. selegiline) or catechol-O-methyltransferase (COMT) are useful adjunctive drugs to potentiate the effect of L-DOPA. However, there are still clinical unmet needs, such as reduction of levodopa-induced dyskinesias (LIDs) and motor fluctuation (e.g. on-off or wearing-off phenomena) after chronic treatment of L-DOPA, non-motor symptoms (e.g. cognitive impairments and affective disorders), as well as lack of substitutes for L-DOPA.

Since 5-HT1A receptor was discovered to be involved in regulating the motor functions such as extrapyramidal motor symptoms, tandospirone has shown antiparkinsonian action in both preclinical studies and clinical trials. Tandospirone improved walking stability and activity of daily life in approximately 40% patients with PD [77]. An eight-week open clinical study revealed that tandospirone exhibited antiparkinsonian effect evaluated by Simpson Angus Extrapyramidal Symptom Scale (SA-EPS) scores [78]. Furthermore, tandospirone had been reported to ameliorate pure akinesia in a patient with resistance to noradrenergic precursor L-threo-DOPS [79]. Along with the clinical studies, numerous preclinical studies have been conducted and shown that tandospirone induced contralateral rotation behaviors in dopaminergic hemilesioned rats evoked by 6-hydroxydopamine (6-OH-DA), ameliorated haloperidol-induced catalepsy and bradykinesia, and reserpine-induced hypolocomotion in dopamine-depleted rats [80–83]. It should be noted that the ameliorative effect of tandospirone in above animal models was nearly fully antagonized by pre/co-administration of WAY-100635, a potent 5-HT1A receptor antagonist, but not haloperidol, a D2 antagonist, indicating that the antiparkinsonian effect of tandospirone is associated with direct activation of 5-HT1A receptor and independent of dopaminergic activity. Intrastriatal injection of 5-HT at doses compatible to those of dopamine (5-HT, 25–100 μg; dopamine, 50–100 μg) caused a contralateral circling behavior in rats, implying that the striatal 5-HT1A receptors played a role in antiparkinsonian effects [84]. Moreover, inactivation of 5-HT neurons by *p*-chlorophenylalanine did not alter the antibradykinesia effect of 8-OH-DPAT [83]. In general, it was the action of postsynaptic 5-HT1A receptor that tandospirone elicited its antiparkinsonian effects. Furthermore, several studies have indicated that there was an increase of releasing glutamate in the regions such as entopeduncular nucleus and striatum as well as an enhancement of Fos expression in the shell and core regions of nucleus accumbens, dorsolateral striatum, and lateral septal nucleus in dopaminergic hemilesioned rats or dopamine-depleted rats [85]. 5-HT1A receptor agonists inhibited the cortico-striatal glutamate pathway and reduced extracellular glutamate levels in the striatum, and tandospirone significantly reduced haloperidol-induced Fos expression in the dorsolateral striatum [86, 87]. Thus, it was proposed that after activating the postsynaptic receptors, tandospirone inhibited cortico-striatal glutamate pathway, reduced the releasing of glutamate and decreased the activity of glutamate neurons and the expression of Fos, resulting in the effect of ameliorating motor dysfunction.

Unlike the antiparkinsonian effect, tandospirone showed only limited effect for alleviating L-DOPA-induced dyskinesia (LID) in several studies. Tandospirone has been tested in 10 patients with LID, and results showed that L-DOPA-induced dyskinesia was considerably alleviated in half of tested patients but Parkinson-like features were slightly worsened in 6 out of 10 patients. In other words, the effect of tandospirone of ameliorating LID was probably at the expense of worsening parkinsonism [88]. In a recent study, tandospirone attenuated L-dopa induced peak abnormal involuntary movements (AIM) scores by about 40% at the highest dose (2.5 mg/kg) but failed to significantly reduce the total AIMs scores in one testing session [89]. In the same study, tandospirone (0.16 mg/kg, 0.63 mg/kg, and 2.50 mg/kg) did not improve the effect of L-dopa in cylinder and rotational behavior test, and decreased the effect of L-dopa in rotarod performance at the highest dose. Given the limited literature, more studies should be conducted to define the definite effect of tandospirone on LID.

Nearly 40% patients with PD also suffer depression (major or minor), and anxiety (e.g. GAD, panic disorder). Thus, depression and anxiety are common in the patients with PD. As stated above, tandospirone is an effective and safe anxiolytic and antidepressant, so it is reasonable to use tandospirone for the treatment of affective disorders in the patients with PD [90, 91].

### Tandospirone in the treatment of schizophrenia

Schizophrenia is a holergasia with unknown etiology and characterized by positive symptoms (e.g. hallucinations, delusion, and excitation), negative symptoms (e.g. apathy, social and emotional withdrawal), cognitive impairments, and mood disturbances (e.g. anxiety and depression) [92, 93]. Classical antipsychotics (e.g. phenothiazine, butyrophenone, and haloperidol) mainly blocking D2 receptor have contributed to control of positive symptoms of schizophrenia but induced severe extrapyramidal side effects (EPS). Since atypical antipsychotics (e.g. clozapine, risperidone, ziprasidone, perospirone, olanzapine, and quetiapine), the second generation of antipsychotics, became the first line treatment for schizophrenia, positive symptoms, negative symptoms, and typical antipsychotics induced EPS have been ameliorated. Nonetheless, there are still clinical unmet in the treatment of schizophrenia, such as cognitive dysfunction, affective disorders, and antipsychotic-induced EPS.

Apart from beneficial effect on affective disorders and EPS demonstrated above, tandospirone also has therapeutic potential for improvement of schizophrenia-associated cognitive deficits. Cognitive functions including memory, executive function and attention are impaired in most patients with schizophrenia, and secondary verbal memory and executive function have been reported to be predominant index for outcome measurement [94, 95]. Some studies have shown that atypical antipsychotics modestly improve cognitive function with various mechanisms, and a 5-HT1A-dependent mechanism may be involved to promote the release of dopamine from the cortex [96, 97]. Thus, it is logical to postulate that 5-HT1A agonists have beneficial effect on improvement of cognitive function. Indeed, numerous clinical studies have shown that chronic adjunctive treatment of tandospirone with typical or atypical antipsychotics significantly improved the cognitive function in patients with schizophrenia. Specifically, a preliminary open-label clinical study revealed the beneficial effect of combined use of tandospirone (30 mg/day) and moderate doses of haloperidol in enhancing attentive function in schizophrenia patients [98]. Subsequently, a similar study with 11 patients with schizophrenia and same number of healthy volunteers as control indicated that the addition of tandospirone with small to moderate doses of haloperidol improved secondary verbal memory in the patients with schizophrenia [99]. In another study, the effectiveness of tandospirone for ameliorating cognitive impairment, especially for executive function and secondary verbal memory, was also confirmed in 26 patients with schizophrenia after tandospirone was added to ongoing treatment with typical antipsychotic drugs for 6 weeks [100]. In two single case reports, tandospirone improved cognitive performance and quality of life when combined with atypical antipsychotics olanzapine or perospirone, respectively [101, 102]. However, it was also reported that acute (60 min) administration of tandospirone impaired explicit verbal memory in healthy volunteers in a dose-dependent manner [103]. The treatment regimen (acute *vs.* chronic) and/or subjects studied (health controls *vs.* schizophrenia patients) may account for the discrepancy between these studies.

Data from animal studies also revealed the superiority of tandospirone in the treatment of schizophrenia. Novel object recognition (NOR) deficits could be induced by subchronic treatment of phencyclidine (PCP), a N-methyl-D-aspartate (NMDA) receptor antagonist, and NOR is an analog of declarative memory and its deficits were often observed in animals and patients of schizophrenia with cognitive impairment [104]. Tandospirone alone or co-treatment with lurasidone for 15 days significantly reversed PCP-induced NOR deficits [105]. There was an increased release of 5-HT in the cortex or 5-HT1A receptor binding potential in the medial- and dorsolateral-frontal cortex, when PCP was acute or subchronic administrated [106, 107]. Similar findings were also confirmed in patients with schizophrenia by postmortem and positron emission tomography [108, 109]. The evidence suggests that tandospirone could reverse NOR deficits, probably related to its 5-HT1A agonist properties, diminishing 5-HT release, and normalizing 5-HT1A receptor binding potential. Besides, tandospirone improved cognitive deficits by monotherapy or in combination with blonanserin, an atypical antipsychotic, assessed by executive function in marmosets using object retrieval with detour (ORD) task [110]. This study also revealed that the D1 receptor agonist SKF-81297 improved the marmosets performance in ORD task. It has also been reported that tandospirone increased the extracellular dopamine level in the prefrontal cortex [60]. Thus, it is possible that tandospirone actives the 5-HT autoreceptor in the raphe nuclei, inhibits the firing of 5-HT neurons, disinhibits the DA neurons, increases the release of DA, and ultimately improves the executive function. Interestingly, augmentation use of tandospirone and haloperidol showed no beneficial advantages in executive function in this study while the combination treatment had shown its beneficial effect on memory function in clinical trial as stated above [98, 99, 110]. Furthermore, another NMDA receptor antagonist, dizocilpine (MK-801), were transiently administrated to the neonatal rats and suppressed stress-induced increment of lactate production, and chronic treatment of tandospirone attenuated the suppressive effect induced by dizocilpine via elevating lactate production [111]. These data indicate that lactate production may play an important role in ameliorating cognitive impairment in schizophrenia.

Study also showed that tandospirone decreased locomotor activity in the rats with or without dizocilpine treatment but the efficacy was only observed with the dose of tandospirone at 5 mg/kg not at the low dose of 0.05 mg/kg, indicating that tandospirone at high dose could be effective to relieve positive symptoms of schizophrenia [112]. It is of interest that this inhibitory effect of tandospirone could be augmented by acute administration of 5-HT1A antagonist WAY 100635, suggesting the underlying mechanism may be associated to its metabolites or other neurotransmitter systems. Tandospirone is metabolized to 1-(2-pyrimidinyl)-piperazine (1-PP) in rodents and humans, and as an α2-adrenoceptor antagonist, 1-PP induced hypolocomotion in mice and rats [113, 114]. Therefore, it is possible that the α2-adrenoceptor-antagonistic effect of 1-PP was responsible for the efficacy of tandospirone in decreasing locomotion. However, in the same study, tandospirone exacerbated dizocilpine-induced prepulse inhibition (PPI) of the acoustic startle response. This phenomenon was observed by measuring the deficits in sensorimotor gating and frequently occurred in patients with schizophrenia. Another study showed that tandospirone suppressed impulsive action in a dose-dependent manner, while higher impulsivity is often observed in the patients with schizophrenia [115]. Moreover, the suppressive effect of tandospirone could not be reversed by WAY100635 either at 0.3 mg/kg or higher dose 1 mg/kg, in contrast, WAY100635 even strengthened the suppressive effect of tandospirone. It is speculated that as a partial agonist for 5-HT1A receptor, tandospirone may also act as an antagonist, however, there is no clear evidence to prove it so far. In summary, tandospirone could be a promising candidate for the treatment of schizophrenia.

## ASSOCIATED MECHANISM OF TANDOSPIRONE IN THE TREATMENT OF CNS DISORDERS

### Activating signal transduction pathway

It is generally believed that tandospirone acts on 5-HT1A receptor in membrane of dorsal raphe nucleus and limbic area to activate G-protein-activated inwardly rectifying K^+^ (GIRK) channels. This process will arouse some changes as follows: on the one hand, hyperpolarization of target neurons by potassium efflux and consequently inhibition of neuronal firing [116]; on the other hand, inactivation of adenylyl cyclase for subsequently inhibition of the cAMP-PKA cascade. Alternatively, tandospirone increases the phosphorylation of extracellular signal-regulated kinase (ERK) in both hypothalamic paraventricular nucleus and the dorsal raphe nucleus, and these responses could be blocked by the 5-HT1A antagonist WAY 100635, suggesting the involvement of mitogen-activated protein (MAP) signal pathway [117]. Study has shown that chronic treatment of tandospirone desensitized 5-HT1A receptor on the dorsal raphe nucleus and hypothalamic paraventricular nucleus but not hippocampus of brain [118], the ERK signal pathway may underlie the difference.

### Elevating dopamine level

**Figure 2:**
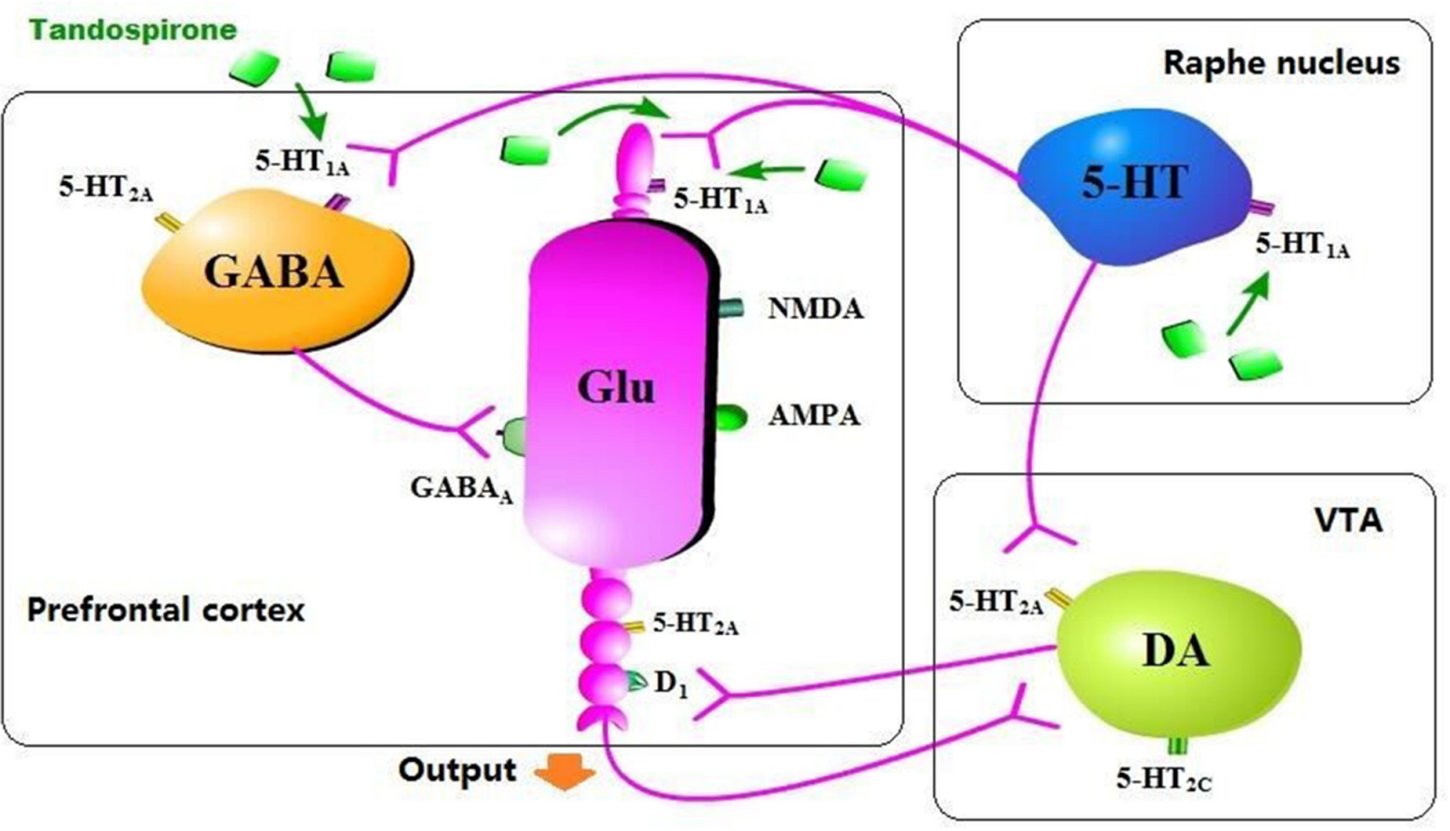
The underlying mechanism of tandospirone in elevating dopamine level Tandospirone activates 5-HT1A receptor in the raphe nucleus or mPFC, directly or indirectly excites and/or disinhibits DA neuron in VTA and increases DA release.

Tandospirone treatment (5 mg/kg) ameliorated the synaptic dysfunction before and after extinction trials in a rat model of juvenile stress [aversive footshock (FS) rat] [47]. Extinction processes contains extinction training and extinction retrieval, which are associated with synaptic efficacy in the hippocampal CA1 and medial prefrontal cortex (mPFC), respectively. Tandospirone increased extracellular dopamine levels in mPFC after extinction retrieval in the non-FS rats (control). Based on the fear extinction study, the effectiveness of tandospirone for the treatment of patients with anxiety especially PTSD, may rely on improving synaptic efficacy associated with extinction processes by elevating dopamine release [47]. Simultaneous administration of tandospirone and fluoxetine significantly increased dopamine release compared to monotherapy in the medial frontal cortex of rats measured by microdialysis. In addition, pre-administration or local perfusion of WAY 100635 suppressed tandopirone-induced increment of extracellular DA. These results suggest that tandospirone elevates extracellular dopamine by stimulating 5-HT1A receptors in mPFC and it may have potential to modulate refractory depression in addition to SSRI [60]. Tandospirone showed beneficial effects on executive function in marmosets in ORD task, and increased extracellular dopamine level in mPFC in previous study [110]. In addition, it is hypothesized that 5-HT1A receptor agonist improves cognitive function in patients with schizophrenia through enhancement of cortical dopamine neurotransmission [119–121]. In the same study, the D1 receptor agonist SKF 81297 improved marmosets’ performance in ORD task [110]. Taken together, tandospirone has beneficial effect on the improvement of cognitive dysfunction in patients with schizophrenia and the possible mechanistic action may be associated with increased dopamine neurotransmission (Figure 2).

### Maintaining hippocampal neurogenesis and synaptic plasticity

Chronic treatment of tandospirone reversed the decrease in the density of doublecortin (DCX)-positive cells in the dentate gyrus of hippocampus both in patients with major depressive disorder and stress-induced social defeat rats [57, 62]. However, no difference was observed between vehicle and tandospirone-treated groups using marker Ki-67 in the animal study [62]. Adult hippocampal neurogenesis had been reported to relate to stress-induced pathophysiology of depressive disorder and one of the mechanistic actions of antidepressants such as SSRI [122, 123]. Moreover, hippocampal neurogenesis plays a role in maintenance of the function of dentate gyrus and hippocampal circuitry, and cognitive function affected by AD [124]. Thus, tandospirone has beneficial effects on anxiety and depressive disorders as well as AD, from the standpoint of hippocampal neurogenesis.

Tandospirone treatment showed no reduction in hippocampal long-term potentiation (LTP) compared to diazepam in mossy fiber-CA3 and perforant path-dentate gyrus synapses, except in Schaffer collateral-CA1 [125]. Hippocampal synaptic plasticity such as LTP is regarded as the electrophysiological basis of synaptic mechanism and the molecular basis of learning and memory [126]. These facts indicate that tandospirone is superior to benzodiazepine in the improvement of learning and memory impairment.

### Normalizing lactate production

**Figure 3:**
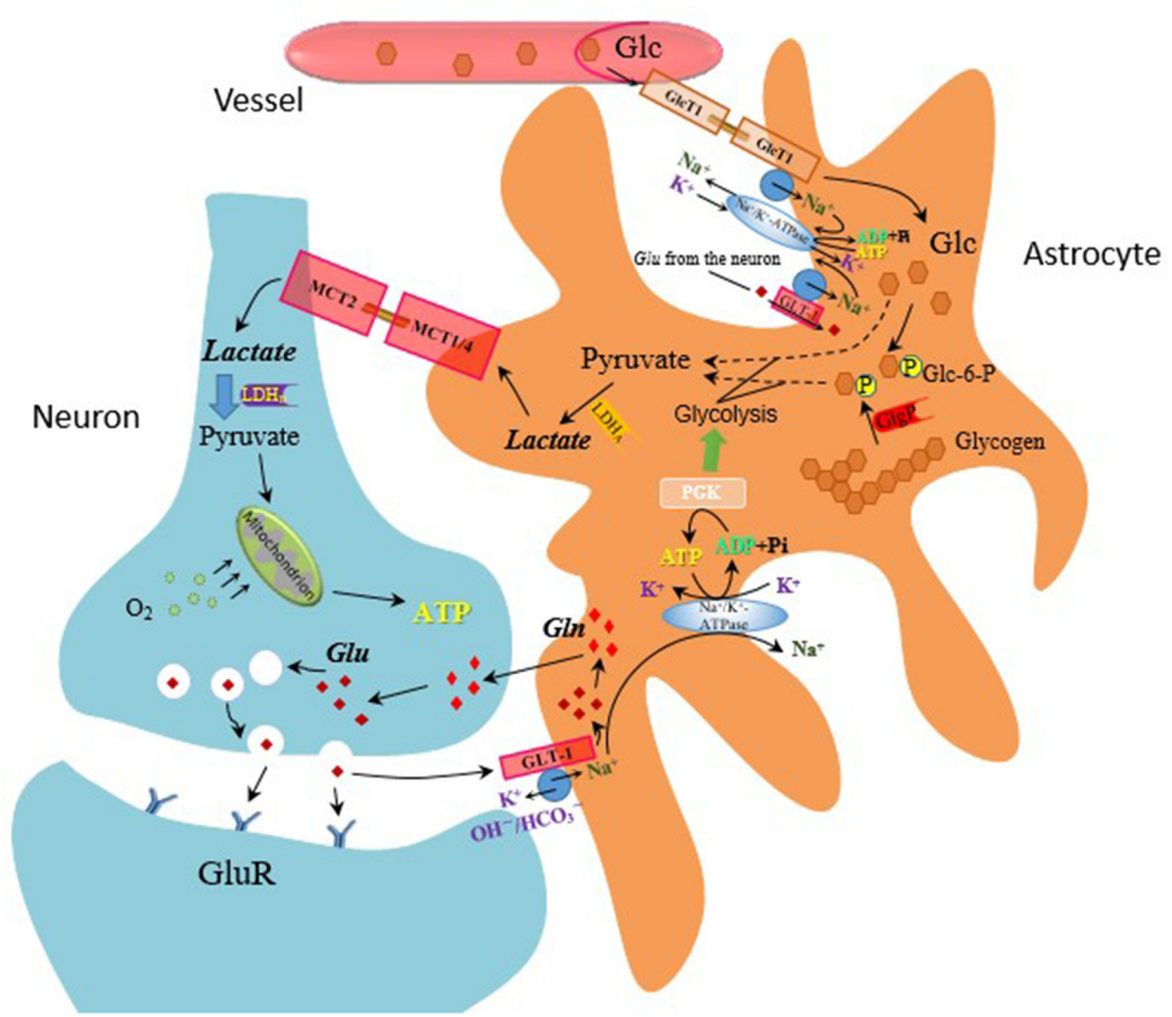
The astrocyte-neuron lactate shuttle hypothesis—mechanism of lactate production on glutamate release Glucose (Glc) from the blood circulation is converted to lactate by astrocytes accompanied by glutamate (Glu) uptake.

Although glucose is the major substrate for energy supply in the brain, lactate gradually shows its significance in energy metabolism in acute neural activation [127, 128]. According to the astrocyte-neuron lactate shuttle hypothesis, glucose supplied from the blood circulation is converted to lactate by astrocytes accompanied by glutamate uptake. Specifically, the activation of nerve cells leads to the release of the neurotransmitter glutamate. Glutamate is actively taken up into astrocytes by glutamate transporters (GLT-1) and is converted into glutamine. The uptake of glutamate into astrocytes increases glucose uptake from surrounding capillaries via glucose transporters and aerobic glycolysis. Lactate is then released to the extracellular space and utilized by activated neurons (Figure 3). More importantly, recent studies suggested that the brain prefers lactate over glucose for energy supply in the state of acute neural activation and lactate has definitely neuroprotective effect [129–131]. Both acute and chronic treatment of tandospirone attenuated footshock stress-induced extracellular lactate concentrations (eLAC) increment in mPFC [63, 132]. Acute treatment of tandospirone reduced the firing rate of 5-HT neurons in the raphe nuclei, subsequently reduced 5-HT release in the mPFC, and consequently provided a negative feedback to the raphe nuclei, which could reduce energy demand in the state of neural activation [13, 132]. On the other hand, chronic administration of tandospirone leads to desensitize the somatodendritic 5-HT1A autoreceptor and normalizes 5-HT releasing. As a 5-HT1A partial agonist, tandospirone also activates postsynaptic receptor, leading to counteracting the increased 5-HT concentrations, resulting in decreasing energy demand in the mPFC. Since lactate is produced in a neural activity-dependent manner, decreased energy demand results in decrease of eLAC in footshocked rats. However, 1-(2-pyrimidinyl)- piperazine (1-PP), a metabolite of tandospirone exerts an α2-adrenoceptor antagonist effect [114] and increases the firing rate of 5-HT neurons, it may compromise the beneficial effect of tandospirone. As stated above, the production of lactate is also mediated by glutamate uptake. In addition, infusion of 5-HT1A agonists into the PFC results in reversing NMDA receptor antagonist-induced glutamate release [133]. Thus, it is speculated that the effect of tandospirone on decreasing footshock-induced increment of eLAC may be mediated by inhibition of glutamate release.

Furthermore, tandospirone reversed the decrease of eLAC induced by transient blockade of NMDA antagonist in response to stress [111]. NMDA receptor antagonists (MK-801 or PCP) led to long-term impairment of cognitive function in schizophrenia in rodents determined by the delayed spatial alteration task and set-shifting task [134, 135]. Besides, the NMDA receptor antagonists also enhance neuronal apoptosis and neuronal degeneration, and reduce spine density in the frontal cortex [136, 137]. These facts suggest that MK-801 in decrease of eLAC level may be due to decreasing the numbers of neurons and/or astrocytes. Besides, systemic administration of 5-HT1A receptor agonists could inhibit the action potentials of GABA neurons, consequently disinhibit glutamate neurons resulting in glutamate release and increment of eLAC [119, 138] (Figure 2). It is putative that the effect of tandospirone on restoration of MK-801-induced eLAC decrease in response to physical stress may be mediated by modulation of glutamatergic neurotransmission via 5HT1A receptors on GABAergic interneurons. Interestingly, a completely opposite result was reported by a study in 2014, which showed that high dose of tandospirone (5 mg/kg) suppressed footshock-induced eLAC elevation in rats treated with MK-801 [139]. One of the explanations for the different results could be the timing and regimen differences with MK-801 administration, e.g. acute vs chronic administration and/or at neonatal vs young adult rats. Taken together, the effects of tandospirone on brain energy metabolism especially in lactate production may be a novel mechanism in the treatment of Schizophrenia.

## CONCLUSION

Tandospirone, a partial 5-HT1A receptor agonist, has been commonly used in the treatment of anxiety disorders, especially in China and Japan. This review provides evidence that tandospirone has beneficial effect in the treatment of other CNS disorders such as Alzheimer's disease, Parkinson's disease, and schizophrenia. Along with better understanding of the associated mechanisms of tandospirone in the treatment of these diseases, it is anticipated that tandospirone could be more frequently utilized in the treatment of the patients with CNS disorders besides anxiety.
